# Assessment of Zoonotic Transmission of *Giardia* and *Cryptosporidium* between Cattle and Humans in Rural Villages in Bangladesh

**DOI:** 10.1371/journal.pone.0118239

**Published:** 2015-02-19

**Authors:** Amimul M. Ehsan, Thomas Geurden, Stijn Casaert, Sonia M. Parvin, Taohidul M. Islam, Uddin M. Ahmed, Bruno Levecke, Jozef Vercruysse, Edwin Claerebout

**Affiliations:** 1 Department of Virology, Parasitology and Immunology, Ghent University, Merelbeke, Belgium; 2 Department of Medicine, Bangladesh Agricultural University, Mymensingh, Bangladesh; Thomas Jefferson University, UNITED STATES

## Abstract

*Giardia* and *Cryptosporidium* are important causes of diarrhoea in Bangladesh. The high prevalence of both parasites in humans and cattle in rural Bangladesh and the common use of water ponds by village inhabitants and their animals suggest a potential for zoonotic transmission. Direct transmission of *Giardia* and *Cryptosporidium* between cattle and their handlers and indirect transmission through water ponds was investigated. Faecal/stool samples were collected from 623 calves and 125 calf handlers in a cross-sectional survey. In two villages, water samples were collected monthly from water ponds and faecal/stool samples were collected monthly from inhabitants and their cattle. *Giardia* cysts and *Cryptosporidium* oocysts were detected in water samples and in faecal/stool samples and positive samples were genotyped, to determine their human or animal origin. The prevalence of *Giardia* and *Cryptosporidium* in calves was 22% and 5% respectively. In calf handlers, the prevalence of *Giardia* and *Cryptosporidium* was 11.2% and 3.2% respectively. Both in the cross-sectional survey and in the longitudinal study in the villages, *G. duodenalis* assemblage E was most prevalent in calves, while in humans assemblage AII, BIII and BIV were found. In cattle, *Cryptosporidium parvum*, *C. bovis* and *C. andersoni* were identified, but no *Cryptosporidium* sequences were obtained from humans. *Giardia* and *Cryptosporidium* were detected in 14/24 and 12/24 water samples respectively. *G. duodenalis* assemblage E and BIV (-like), as well as *C. andersoni* and *C. hominis* were identified. Although the presence of *Giardia* and *Cryptosporidium* in both water ponds suggests that water-borne transmission of *Giardia* and *Cryptosporidium* is possible, the genotyping results indicate that there is no significant direct or indirect (water-borne) transmission of *Giardia* between cattle and people in this area of rural Bangladesh. No conclusions could be drawn for *Cryptosporidium*, because of the low number of sequences that were obtained from human and water samples.

## Introduction

Diarrheal diseases are a serious public health problem, affecting mainly children in developing countries [1, 2]. Several bacterial, viral and parasitic agents are responsible for diarrheal disease, including *Giardia* and *Cryptosporidium* [3, 4]. Susceptible hosts are infected either through contact with an infected host or indirectly by oral intake of infective (oo)cysts through contaminated water or food. Among the eight assemblages (A-H) of *Giardia duodenalis*, assemblages A and B are found in both humans and animals. The occurrence of the same species/genotypes in human and non-human hosts (dogs, cats, livestock and wildlife) in the same geographical areas supports the potential for zoonotic transmission [5]. Most cases of human cryptosporidiosis are due to infections with the human specific *C*. *hominis* or the zoonotic *C*. *parvum* [6]. Other *Cryptosporidium* species have also been detected in humans, although less frequently [7–9]. Current evidence indicates that ruminants are a reservoir of zoonotic *Cryptosporidium* from where humans get infected by contaminated food and water or through direct contact with livestock, for example animal handlers [10, 11].

In Bangladesh, both *Giardia* and *Cryptosporidium* have been associated with diarrhea [12–15]. In cases of giardiosis, mainly *G*. *duodenalis* assemblage A was associated with diarrhea [14, 15]. *Cryptosporidium hominis* as well as *C*. *parvum* were identified in patients with diarrhea [15]. The studies mentioned above were all performed in Dhaka, one of the fastest growing megacities of the world. The presence of overpopulated slums and the pollution of neighbouring rivers with untreated wastewater [16, 17] could facilitate (water-borne) human-to-human transmission.

In rural Bangladesh, *Giardia* infections have also been associated with diarrhea in children [18]. In rural villages of Bangladesh, there are many smallholder cattle farms consisting of 1–5 animals for milk and draft purposes. A previous study found a high prevalence of *Giardia* and *Cryptosporidium* in cattle in Bangladesh [19]. Young calves are considered as a reservoir for these parasites, and transmission of *Giardia* and *Cryptosporidium* from cattle to cattle handlers has been suggested in Bangladesh and India [19, 20]. In addition, people living in rural villages have no or limited access to purified tap water and therefore rely on surface water from ponds or tube wells for their water supply. As water from tube wells is often contaminated with arsenic [21], surface water is often the only alternative. These small ponds are used for daily household purposes (*e*.*g*. swimming, washing of cloths, bathing cattle, cleaning vegetables, fish, utensils etc.). Faeces from cattle that are kept alongside of these ponds are often washed and drained into the ponds. Some households use latrines of which the outlets flow into the ponds. The above-mentioned scenarios indicate that there is a potential for water-borne transmission of *Giardia* and *Cryptosporidium*. However, no studies are available on transmission of *Giardia* and *Cryptosporidium* in these settings.

The aim of this study was to investigate potential direct transmission of *Giardia* and *Cryptosporidium* between cattle and their handlers and indirect water-borne transmission of *Giardia* and *Cryptosporidium* between the inhabitants and their animals in rural villages in Bangladesh.

## Materials and Methods

### Sampling design

***Giardia* and *Cryptosporidium* in calves and their handlers**. A cross-sectional study was carried out to investigate the prevalence of *Giardia* and *Cryptosporidium* in calves (during March and April, 2009 and from March, 2012 to February, 2013) and their handlers (from July, 2012 to February, 2013) in Sadar Upazilla, Mymensingh District, a rural area in the Northeast of Bangladesh. Since there is no livestock database in Bangladesh, the first step of the sampling process was the digitisation of the map of Mymensingh district using ArcView Version 3.2 (Environmental Systems Research Institute, Inc. Redlands, California) which was previously carried out [22]. All 13 unions (sub Upazilla) namely Baera, Bhabkhali, Char Ishwardia, Char Nilaxmia, Khagdahar, Kustia, Sirta, Ashtadhar, Bobar Char, Dapunia, Ghagra, Paranganj and Sadar Upazilla, Mymensingh district were selected. One geographical coordinate was randomly selected from 10 previously selected coordinates in each union using the random number generation function in Microsoft Excel and located by a hand held GPS reader. Livestock farmers within a 0.5 km radius of the selected point were informed about this study [23]. To encourage livestock farmers to participate, free anthelmintics and vitamin-mineral premix were supplied to their animals when sampling took place. Rectal faecal samples from 30–40 calves aged between 5 days and 6 months were collected from each union. The specimens (n = 623) were labeled as par ear tag of individual calves and immediately transported to the laboratory of the Department of Medicine, Bangladesh Agricultural University, Mymensingh. The calf handlers (n = 125) were provided with sterile wide-mouthed plastic containers for collection of stool specimens. All specimens were kept at 4°C and processed within 24 hours of collection. All animals and individuals were healthy at the time of sampling.

**Fig 1:**
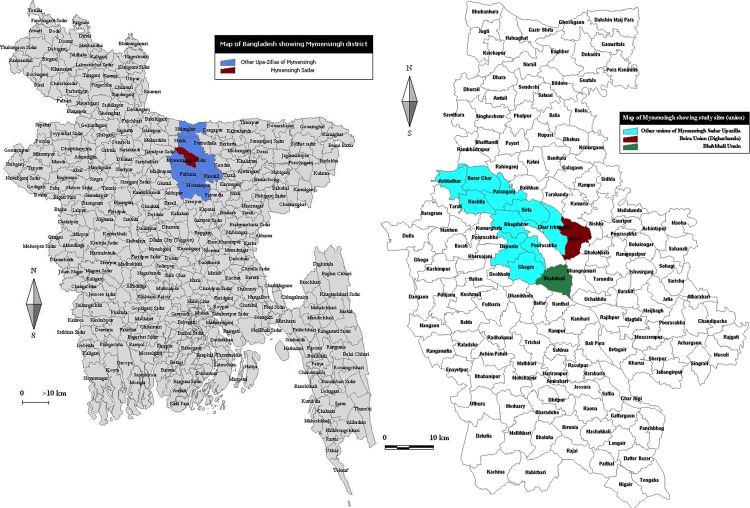
Study area. Map of Bangladesh showing Mymensingh district, Sadar Upazilla and unions of Sadar Upazilla. Left: map of Bangladesh showing Mymensingh district (blue) with Sadar Upazilla (red); Right: Sadar Upazilla (cyan) with the two sampled villages (red = Digarkandha and green = Bhabkhali).

**Water-borne transmission of *Giardia* and *Cryptosporidium* in two rural villages**. To investigate potential water-borne transmission of *Giardia* and *Cryptosporidium*, samples were collected from water ponds in two villages, as well as from inhabitants from households using these ponds and from their cattle. These two villages, Bhabkhali and Digarkandha, are situated in Mymensingh District (Fig. 1). Both villages have a population of about 4,000 persons in 800 households. Almost every household keeps 1–5 cattle for milk and draft purposes alongside the water ponds. These small ponds are used for household purposes and as main sources of water supply. Like in most of rural Bangladesh, the villages rely mainly on hand-pumped water for drinking and on surface water sources for other domestic and personal purposes. As hand-pumped tube wells are often contaminated with arsenic, villagers rely on surface water as an alternative. The ponds were selected on the basis of availability of water in all seasons and frequent use of the ponds by surrounding village people and their animals. A grab water sample (15L) was collected from one pond in each village every month at the same place from March, 2012 to February, 2013 and all samples (n = 24) taken immediately to the laboratory.

Rectal faecal samples from 10 cattle and 10 human stool samples were randomly collected without any age and sex restriction per month in each village from March, 2012 to February, 2013. In March 2012, exceptionally 20 cattle and 20 humans were sampled instead of 10, resulting in a total of 130 human and 130 cattle samples. All sampled individuals and animals were healthy at the time of sampling. The specimens were labelled as par ear tag of individual cattle and name of the person and immediately transported to the laboratory. All specimens were kept at 4°C and processed within 24 hours of collection.

### Detection of *Giardia* cysts and *Cryptosporidium* oocysts in faecal/stool samples

Faecal samples were examined using a quantitative immunofluorescence assay (IFA; Merifluor *Cryptosporidium*/*Giardia* kit; Meridian Diagnostics Inc.) [24]. In short, 1g of faecal sample was suspended in distilled water and strained through a layer of surgical gauze to withhold large debris. After sedimentation for 1h and centrifugation at 3000*g* for 5 min, the sediment was resuspended in distilled water up to a volume of 1ml. After thorough vortexing, an aliquot of 20μl was pipetted onto a treated IFA-slide. After staining the slide, as instructed by the manufacturer, the entire smear was examined at a 400x magnification under a fluorescence microscope. The number of *G*. *duodenalis* cysts per gram faeces was obtained by multiplying the total number of cysts on the smear by 50. A sample was considered to be positive when at least one *G*. *duodenalis* cyst was found on the IFA slide.

### Detection of *Giardia* cysts and *Cryptosporidium* oocysts in water samples

A protocol was optimised to detect *Giardia* and *Cryptosporidium* in water samples, based on the USEPA method 1623 [25]. To validate the protocol, spiking experiments were performed with water samples collected from Diagarkandha and Bhabkhali in March and November 2012. Water samples were spiked with 100 inactivated *Giardia* cysts and 100 inactivated *Cryptosporidium* oocysts, permanently labeled with red fluorescent dye (ColorSeed, BTF Pty Ltd., North Ryde, Australia). The ColorSeed (oo)cysts were added to each water sample prior to filtering to estimate the percent recovery of (oo)cysts according to the manufacturer’s instructions.

Both spiked (15L) and non-spiked water samples (15L) were filtered through Filta-Max filters (IDEXX Laboratories, Inc., Westbrook, ME, USA) with the aid of a motorized peristaltic pump with recommended flow rates of 2L/min. The Filta-Max filters were washed with the Filta-Max manual wash station for elution of the filters following the manufacturer’s instructions. The eluate was centrifuged and the sediment was used for immunomagnetic separation (IMS) of the (oo)cysts. Cysts and oocysts in the sediment were purified by IMS using *Giardia* and *Cryptosporidium* specific antibody-coated magnetic beads according to the manufacturer’s protocol (Dynabeads GC-Combo, Invitrogen Dynal, A.S., Oslo, Norway). IMS-purified cysts and oocysts were stained on well slides by fluorescein isothiocyanate (FITC)-conjugated anti-*Giardia* and anti-*Cryptosporidium* MAbs FITC conjugated monoclonal antibodies (EasyStain, BTF Pty Ltd. Australia). Slides were examined using a Leica Leitz DMRB fluorescence microscope. The well surface was scanned at 200 or 400 times magnification for *Giardia* cysts and *Cryptosporidium* oocysts using a FITC/TRITC fluorescence filter (450–590 nm Chroma technology corp.) and Texas Red fluorescence filter (530–585 nm, FT600, LP615) to distinguish ColorSeed (oo)cysts (which fluoresce red with the Texas Red filter) from natural (oo)cysts (which fluoresce bright green with the FITC filter). *Giardia* cysts and *Cryptosporidium* oocysts were identified and counted based on their size, morphology and fluorescence. Results were expressed as recovery percentage for spiked (oo)cysts and as count per L for naturally occurring (oo)cysts. Slides containing (oo)cysts were kept at 4°C for DNA extraction.

### DNA extraction and molecular analysis

DNA was extracted from non-spiked water samples and faecal samples from cattle and humans that were positive for *Giardia* and/or *Cryptosporidium* in immunofluorescence microscopy. Genomic DNA was extracted from water sediment and from faecal/stool samples using the QIAamp Stool Mini Kit (Qiagen GmbH, Hilden, Germany) and from (oo)cysts that were scraped from the microscope slides from water samples by the QIAamp DNA Mini Kit (Qiagen GmbH, Hilden, Germany) according to the manufacturer’s instructions, incorporating an initial step of 3 freeze-thaw cycles (freezing in liquid nitrogen for 5 min and heating at 95°C for 5 min) in the protocol to maximize disruption of (oo)cysts.

For the identification of *Giardia* the β-giardin gene [26] was used in 2-step nested PCR. In addition, the triose phosphate isomerase (TPI) gene was used for assemblage-specific amplification of *Giardia*, [27,28,29]. Previously described PCR protocols were used to amplify the 18S rDNA gene [30,31] and the 70 kDa heat shock protein (HSP-70) gene [32] of *Cryptosporidium*. For subgenotyping of *C*. *parvum* and *C*. *hominis* positive samples, the 60kDa glycoprotein (GP60) was targeted [33]. For all PCR reactions, negative (PCR water) and positive controls (genomic DNA) were included. The PCR products were visualized in agarose gel (1.5%) stained with ethidium bromide under UV light. PCR products were fully sequenced by the BIG Dye Terminator V3.1 Cycle sequencing Kit (Applied Biosystems). Sequencing reactions were analysed on a 3100 genetic Analyzer (Applied Biosystems) and assembled with the program Seqman II (DNASTAR, Madison WI, USA). To determine the subgenotype the fragments were aligned with homologous sequences available in the GenBank database, using MegAlign (DNASTAR, Madison WI, USA).

### Statistical analysis

The χ^2^-test was used to compare infection rates of *Giardia* and *Cryptosporidium* in calves and humans between the wet season (mid April-mid October, mean rainfall 278 mm, mean temperature 29°C) and the dry season (mid October-mid April, mean rainfall 42 mm, mean temperature 23°C). Mean (oo)cyst counts in water samples from both villages were compared between seasons using a related-samples Wilcoxon signed rank test. All analyses were done using the SPSS statistical programme. Values of p < 0.05 were considered statistically significant.

### Ethics statement

This study was approved by the Ethical Committees of the University Hospital, Ghent University (EC/2012/604) and Mymensingh Medical College, Bangladesh (MMC/EC/97). All subjects provided written informed consent, and a parent or guardian of any child participant provided informed consent on their behalf.

Animal care was approved by the Ethical Committee of the Faculty of Veterinary Science, Bangladesh Agricultural University, Mymensingh (FVS/EB/2012/01), according to the Animal Experimentation Ethics Committee (AEEC) guidelines of the International Centre for Diarrhoeal Disease Research, Bangladesh and the Prevention of Cruelty to Animals Act, 1890 (Act No. XI 1890). Verbal consent of animal owners was obtained prior to the collection of fecal samples.

## Results

### *Giardia* and *Cryptosporidium* in calves and calf handlers

A total of 623 faecal samples from calves were collected during the study period. The prevalence of *Giardia* was 21.7% (95% Confidence Interval (CI): 18–25%), with a mean cyst excretion of 55,038 cysts per gram (CPG) in infected animals (range 50–250,000 CPG). The prevalence of *Cryptosporidium* was 5.0% (95% CI: 3.7%-6.7%) with an average excretion of 1,534 oocysts per gram (OPG) (range 50–25,000 OPG). *Giardia* and *Cryptosporidium* infection rates were not significantly different between seasons. In the wet season, 21.1% of calves were positive for *Giardia* (83/393), compared to 22.6% (52/230) positive calves in the dry season (χ^2^ = 0.236, d.f. = 1, p > 0.05). Similarly, 4.8% (19/393) calves were positive for *Cryptosporidium* in the wet season, compared to 5.2% (12/230) *Cryptosporidium* positive calves in the dry season (χ^2^ = 0.004, d.f. = 1, p > 0.05). *Giardia* sequences were obtained from 60 out of 135 samples that were positive in IFA. The molecular characterization indicated a high prevalence of *G*. *duodenalis* assemblage E (47/60, NCBI accession numbers KJ188040-KJ188084, KJ363342-KJ363377) in calf samples. Other *G*. *duodenalis* assemblages found were AI (10/60, NCBI accession numbers KJ188032-KJ188039, KJ363378, KJ363379) and B (3/60, NCBI accession numbers KJ363380-KJ363382), often in mixed infections with assemblage E (5/60). Genotyping of *Cryptosporidium* positive samples (n = 5) revealed the presence of *C*. *parvum* (2/5, NCBI accession number KJ363386), *C*. *bovis* (1/5, NCBI accession number KJ363383) and *C*. *andersoni* (2/5, NCBI accession numbers KJ363384, KJ363385). Both *C*. *parvum* samples that were successfully amplified in the GP60 PCR belonged to sub type IIdA16G1 (NCBI accession numbers KJ363387, KJ363388).

*Giardia* cysts were detected in 14 out of 125 calf handler’s stool samples (11.7%, 95% CI: 5.7–16.7%) with a mean cyst excretion of 33,168 CPG (range 50–250,000 CPG) and *Cryptosporidium* oocysts in 4 samples (3.3%, 95% CI: 1.1%-6.3%), with an average excretion of 275 OPG (range 50–450 OPG). Out of 14 positive samples, 6 samples were successfully sequenced for *Giardia*. Assemblages AII (2/6, NCBI accession numbers KJ188085, KJ188091), BIII (1/6, NCBI accession number KJ188086), BIV (2/6, NCBI accession numbers KJ188087, KJ188094) or a combination of AII, BIII and BIV (1/6, NCBI accession numbers KJ188088, KJ188089, KJ188092, KJ188093) were detected. None of the 4 *Cryptosporidium* samples could be amplified in the PCR reactions.

### *Giardia* and *Cryptosporidium* in water ponds and faecal samples from village inhabitants and their cattle

**Water samples**. Slightly higher recovery rates were obtained for *Giardia* than for *Cryptosporidium*, which is in agreement with previous observations [25, 34]. Mean recovery percentages (with standard deviation) for *Giardia* and *Cryptosporidium* were 41±6% and 40±2%, respectively. Recovery rates for both parasites met the standards set by USEPA, 2005.

A total of 24 water samples (12 from Bhabkhali and 12 from Digarkandha) were collected over a period of 1 year. *Giardia* cysts were detected in 14/24 samples, whereas *Cryptosporidium* oocysts were detected in 12/24 water samples. Both ponds were contaminated with *Giardia* and *Cryptosporidium*. *Giardia* cyst counts in positive samples ranged from 0.1/L to 6.5/L in Bhabkhali and between 0.4/L and 11.3/L in Digarkandha. *Cryptosporidium* oocyst counts varied between 0.3/L and 0.7/L and between 0.2/L and 1.1/L in Bhabkhali and Digarkandha, respectively. Although (oo)cyst counts in Digarkandha appeared to be higher in the wet season, these differences were not statistically significant (p > 0.05) (results not shown).

Samples that contained at least one (oo)cyst detected by IMS-IFA were selected for genotyping. A total of 11 positive water samples for *Giardia* (6 from Bhabkhali and 5 from Digarkandha) and 9 samples for *Cryptosporidium* (4 from Bhabkhali and 5 from Digarkandha) were selected for PCR. *Giardia* sequences were obtained from 5 positive water samples. Most sequences belonged to assemblage E (n = 4; 2 from each village). One sample from Digarkandha could not be differentiated between assemblage BIV and BIV-like and was possibly a mixed infection. *C*. *hominis* (NCBI accession numbers KJ363330, KJ363331) and *C*. *andersoni* (NCBI accession number KJ363329) were identified in 2 and 1 samples from Digarkandha respectively. No successful sequence for *Cryptosporidium* was obtained from Bhabkhali water samples.

**Cattle samples**. The *Giardia* infection rate in cattle in Digarkandha was 13.3% (95% CI: 7.3%-19.4%), with a mean cyst excretion in positive animals of 22,075 CPG (range 50–125,000 CPG), compared to 5.0% (95% CI: 1.1%-8.9%) with a mean cyst excretion of 900 CPG (range 50–3750 CPG) in Bhabkhali. *Cryptosporidium* infection rates were 9.2% (95% CI: 4.0%-14.3%) with a mean excretion of 150 OPG (range 50–600 OPG) and 5.0% (95% CI: 1.1%-8.9%) with a mean excretion of 8417 OPG (range 50–35,000 OPG) in Bhabkhali and Digarkandha, respectively. No significant associations between season and infection rates were observed (p > 0.05) (Table 1).

**Table 1:** Seasonal occurrence of Giardia and Cryptosporidium infections in cattle in Bhabkhali and Digarkandha, expressed as percentage and number of investigated samples that were positive.

Season	Bhabkhali		Digarkandha	
	*Giardia* (%)	*Cryptosporidium* (%)	*Giardia* (%)	*Cryptosporidium* (%)
Wet	3.3 (2/60)	10.0 (6/60)	15.0 (9/60)	5.0 (3/60)
Dry	5.7 (4/70)	7.1 (5/70)	10.0 (7/70)	4.3 (3/70)

A total of 22 positive samples for *Giardia* were selected for PCR (6 from Bhabkhali and 16 from Digarkandha). Most sequences belonged to assemblage E (n = 7; 1 from Bhabkhali and 6 from Digarkandha, NCBI accession numbers KJ363394-KJ363397 and KJ363338-KJ363341). In Digarkandha, one sample was identified as AI (NCBI accession number KJ363336) and one sample had mixed infections of assemblages AI and B (NCBI accession numbers KJ363393, KJ363337). Out of 17 samples that were positive for *Cryptosporidium*, *C*. *andersoni* was detected in one sample in each village (NCBI accession numbers KJ363327, KJ363328) and *Cryptosporidium* horse genotype in one sample from Bhabkhali (NCBI accession number KJ363326).

**Human samples**. Infection rates with *Giardia* and *Cryptosporidium* were similar in inhabitants of both villages. *Giardia* infection rates were 11.7% (95% CI: 9.3%-22.4%) with a mean excretion of 39,950 CPG (range 50–400,000 CPG) and 15.8% (95% CI: 9.3%-22.4%) with a mean excretion of 67,329 CPG (range 50–1,000,000 CPG) in Bhabkhali and Digarkandha, respectively. *Cryptosporidium* was detected in 6.7% (95% CI: 2.2%-11.1%) of human stool samples in Bhabkhali (mean 11,638 OPG, range 50–80,000 OPG) and 5.0% (95% CI: 1.1%-8.9%) of human samples in Digarkandha (mean 325 OPG, range 50–1000 OPG). Although *Giardia* and *Cryptosporidium* infection rates appeared to be higher in the rainy season in both villages (Table 2), differences between seasons were only statistically significant for *Cryptosporidium* in Bhabkhali (χ^2^ = 6.03, d.f. = 1, p < 0.05) and *Giardia* in Digarkandha (χ^2^ = 6.79, d.f. = 1, p < 0.01).

**Table 2:** Seasonal occurrence of Giardia and Cryptosporidium infections in humans in Bhabkhali and Digarkandha, expressed as percentage and number of investigated samples that were positive.

Season	Bhabkhali		Digarkandha	
	*Giardia* (%)	*Cryptosporidium* (%)	*Giardia* (%)	*Cryptosporidium* (%)
Wet	15.0 (9/60)	11.7 (7/60)	23.3 (14/60)	6.7 (4/60)
Dry	7.1 (5/70)	1.4 (1/70)	7.1 (5/70)	2.9 (2/70)

A total of 33 positive samples for *Giardia* were selected for PCR (14 from Bhabkhali and 19 from Digarkandha). *Giardia* sequences were obtained from 6 positive samples (2 from Bhabkhali and 4 from Digarkandha). All sequences belonged to assemblage BIII (n = 3; all from Digarkandha, NCBI accession numbers KJ363334, KJ363390, KJ363391), BIV (1 sample from Bhabkhali, NCBI accession number KJ363335) or a mixed infection of assemblage BIII and BIV (1 in each village, NCBI accession numbers KJ363332, KJ363333, KJ363389, KJ363392). No PCR products were obtained for *Cryptosporidium*.

## Discussion

The prevalence of *Giardia* in calves was higher compared to *Cryptosporidium* in the cross-sectional study. A possible reason was the age range of the calves, as *Cryptosporidium* infection rates are typically highest in calves younger than one month [35, 36] while a large proportion of the calf population in the present study was older than one month. It should be noted that the estimated prevalence in the cross-sectional study could have been affected by intermittent shedding of (oo)cysts, as all subjects were only sampled once.

Higher infection rates in calves were previously observed in the rainy season and in autumn in Bangladesh, India and Pakistan [19, 36–38]. The warm and humid conditions may favour survival of (oo)cysts, and heavy rainfall and flooding may facilitate transmission of the infections. Although in our study infection rates in cattle were not significantly different between the wet and the dry season, a seasonal pattern in *Giardia* and *Cryptosporidium* infections was observed in the village inhabitants. Similarly, higher *Cryptosporidium* infection rates in human patients during the rainy season have been reported previously in Bangladesh [12].

A significant association between the occurrence of *Cryptosporidium* and *Giardia* infections in calves and calf handlers has been documented in previous studies in Bangladesh and in West Bengal, India [19, 20]. However, in this study no evidence was found of direct zoonotic transmission between calves and their handlers. The cattle handlers were almost exclusively infected with *G*. *duodenalis* assemblage AII and BIII, while the calves were predominantly infected with the hoofed livestock-specific assemblage E, and to a lesser extent with assemblage AI. The other assemblages that were occasionally found in cattle (BIV, BIV-like) are also found predominantly in animals [39]. Furthermore, mainly bovine *Cryptosporidium* species were identified in the calves, although two samples belonged to a *C*. *parvum* subtype that has also been reported in human cases (IIdA16G1).

In both villages, water samples from the ponds were frequently contaminated with *Cryptosporidium* and *Giardia*. However, since no viability staining was performed on the detected (oo)cysts, it cannot be excluded that some of the detected (oo)cysts were dead.

No evidence for zoonotic transmission of *Cryptosporidium* and *Giardia* between cattle and humans could be found in these environments with high potential for zoonotic transmission. In both villages, cattle were carrying predominantly animal-specific parasite species (*C*. *andersoni*, *G*. *duodenalis* assemblage E). Although the zoonotic assemblage AI was also detected in a few bovine samples, this assemblage is mostly found in animals [39], and was not identified in any human sample in Bhabkhali or Digarkandha. Similarly, Laishram et al. [40] did not find assemblage AI in children and adults in neighboring India. Only assemblage BIII and BIV were identified in the human population in these villages. Although these assemblages can also infect animals [39], they were not detected in any of the cattle samples. Together with the presence of *C*. *andersoni*, *C*. *hominis* and *G*. *duodenalis* assemblage E and (less frequently) BIV (-like) in the water samples, these data suggest that water-borne transmission of *Cryptosporidium* and *Giardia* may occur in Bhabkhali and Digarkandha, but that (water-borne) transmission cycles are predominantly within host species.

## Conclusions

The present study demonstrated the presence of *Giardia* and *Cryptosporidium* in cattle and people in a rural area of Mymensingh, Bangladesh. Water ponds in rural villages were also contaminated with *Cryptosporidium* and *Giardia*, and may facilitate water-borne transmission of these parasites. However, no evidence was found of zoonotic transmission of *Giardia* from cattle to humans, either directly (between cattle and their handlers) or indirectly through the water ponds, suggesting that (water-borne) transmission cycles of *Giardia* in this area are predominantly within host species. Because of the low success rate of *Cryptosporidium* genotyping in human and water samples, no conclusions can be made about transmission patterns for this parasite.
